# Histopathological Examination of Lung Necropsy of 11 Patients Who Died Due to COVID-19: A Case Series

**DOI:** 10.30699/IJP.2023.2008773.3153

**Published:** 2023-12-29

**Authors:** Bahram Nikkhoo, Karim Naseri, Ramyar Rahimi Darehbagh, Mehrdad Habiby, Bahar Moasses-Ghafari

**Affiliations:** 1 *Department of Pathology, School of Medicine, Kurdistan University of Medical Sciences, Sanandaj, Iran *; 2 *Department of Anesthesiology, School of Medicine, Kurdistan University of Medical Sciences, Sanandaj, Iran *; 3 *Student Research Committee, Kurdistan University of Medical Sciences, Sanandaj, Iran *; 4 *Department of Radiology, School of Medicine, Kurdistan University of Medical Sciences, Sanandaj, Iran*

**Keywords:** COVID-19, Iran, Pathology, Necropsy, Lung

## Abstract

COVID-19 is known to present with acute respiratory distress syndrome pathological manifestations. Studies have shown that patients with COVID-19 can develop diffuse alveolar damage, acute bronchopneumonia, necrotic bronchiolitis, and viral pneumonia.

In this study, we investigated 11 cases. Needle necropsies of 11 patients, hospitalized at Tohid and Kowsar hospitals of Kurdistan University of Medical Sciences, with a positive antemortem SARS-CoV-2 (COVID-19) real-time PCR test, were fixated within 3 hours after death in the negative-pressure isolation morgue. The participants included six men (54%) and five women (46%) with a mean age of 73.82±10.58 (52–86) years old. The average hospitalization was 14.27±15.72 days. The results showed interstitial lymphocytic pneumonitis in most of the cases, varied from mild to moderate and up to severe in some cases. In 7 cases, anthracosis was noted, while one case demonstrated anthracosis with fibrosis. The hyaline membrane was reported in two patients. In one case, severe interstitial lymphocytic pneumonia with intra-alveolar exudate with organization, lithiasis, bronchiolitis pattern (BOOP), intra-alveolar hemorrhage, and mild fibrosis were seen. As a result, it is suggested to keep an eye on these pathologies in management of the severe cases of COVID-19 infection.

## Introduction

Respiratory failure is the ultimate mechanism of death in most patients with severe Coronavirus 2019 (COVID-19) infection. Patients with COVID-19 develop respiratory failure, hypoxemia, and acute bilateral pulmonary infiltration associated with acute respiratory distress syndrome (ARDS). Respiratory failure in COVID-19 may indicate a novel pathology (1, 2).

The available literature currently shows that COVID-19 presents with a known histopathology, which falls into the spectrum of acute respiratory distress syndrome pathological manifestations. However, these pathological features seem to be unclear due to the lack of detailed studies. Therefore, an assessment of the histopathologic events in a spectrum of COVID-19 cases has been focused and the specific interventions for their treatment could highlight the preventive measures, which are mostly associated with less mortality and better clinical outcomes (1).

SARS-CoV-2 infection can lead to various multi-organ injuries, mostly affecting the lungs, heart, and kidneys. Acute diffuse alveolar damage (D.A.D.) and micro-pulmonary thrombosis have been reported in histological examination of the most of patients. Some studies have shown that patients with COVID-19 develop diffuse alveolar damage and commonly develop acute bronchopneumonia and aspiration pneumonia, necrotic bronchiolitis, and viral pneumonia (1-3). Some case studies have also reported a spectrum of pathologies such as interstitial pneumonia, intravascular and extravascular fibrin depositions, hemorrhage and infarcts, and interstitial lymphocytic pneumonitis with alveolar fibrin deposits but no hyaline membrane (4, 5).

However, despite some limited case series studies, there is not enough evidence to highlight the pathology behind the COVID-19 infection, and further studies seem necessary to shed more light on this matter. This study is a case series that investigated 11 cases along with their medical history, duration of their hospitalization, management, and post-mortem necropsy histopathology features.

## Material and Methods

**Patient's Selection and Necropsy Procedures**

This study included eleven patients hospitalized at Kurdistan University of Medical Sciences Tohid and Kowsar hospitals who died due to severe COVID-19 disease manifestations; for all patients, written informed consents were collected from their family members for use of their post-mortem tissue samples for this research. The patients' COVID-19 infection has been confirmed through antemortem SARS-CoV-2 real-time polymerase chain reaction (PCR) by 2019 CoV real-time RT-PCR assay for virus detection designed by C.D.C. in both hospitals. Needle necropsy from the fifth to sixth intercostal spaces in the anterior mid-axillary line from the superior ridge of the coastal bones of the patient cadavers was taken within three hours post-mortem in the negative-pressure isolation mortuary of both hospitals. According to the World Health Organization guidelines, full health protocols and safety protection measures were applied. The samples were then stored in formalin-filled containers and transferred to the pathology lab for further analysis. The Ethics Committee of Kurdistan University of Medical Sciences license with an ethics code I.R. M.U.K.REC.1399.024 was obtained before the study.

**Histologic Examination**

There were containers filled with 10% neutral buffered formalin labeled with the patient's name, hospital, and admissions ward in which the necropsy materials of organs were placed. Then the tissues were processed using standard procedures, including paraffin embedding, microtome sectioning, and hematoxylin-and-eosin staining. The patients' information is presented in Table 1.

**Table 1:** Patient characteristics, comorbidities, and select initial laboratory findings

Case	Age	Sex	Comorbidity	Hospitalization (Day)	Hospitalization in I.C.U. ward	Hospitalization inward	Intubation	Cr	L.D.H.	Anticoagulation	BUN	ESR
1	80	M	C.V.A.	12	6	6	Yes	1	1011	positive	-	
2	72	F	-	5	3	2	No	2	22	positive	-	
3	85	F	COPD	8	7	1	Yes	1	949	negative	45	13
4	52	F	Hypertension	24	8	16	Yes	7	575	negative	74	
5	80	M	-	8	0	8	No	1	630	positive	45	50
6	85	M	Cirrhosis	2	0	2	Yes	3	-	negative	72	
7	67	M	C.V.A.	11	8	3	Yes	5	336	positive	81	52
8	86	F	Hypertension/ CKD	4	0	4	Yes	7	674	positive	67	40
9	70	M	Hypertension	54	35	19	Yes	1		positive	7	38
10	71	F	Single kidney	28	28	-	Yes	2	564	negative	58	
11	64	M	RA/COPD/ Hypertension	1	1	0	No		-	positive	-	

**Data Analysis**

The collected data were presented using descriptive statistical techniques. Stata16.0 was used to perform all statistical analyses (StataCorp, College Station, TX).

## Case Presentation

The patients were referred to the hospital based on typical COVID-19 respiratory complaints such as fever, tachypnea, dyspnea, respiratory distress, limb pain, and, in some cases, gastrointestinal symptoms. They were suspected and tested for SARS-CoV-2 RT-PCR. They received COVID-19 guideline treatments after the results of the test were positive before they died due to the severity of the COVID-19. The details about the hospitalization of the patients are as follows:

**Case 1**

**Fig. 1:**
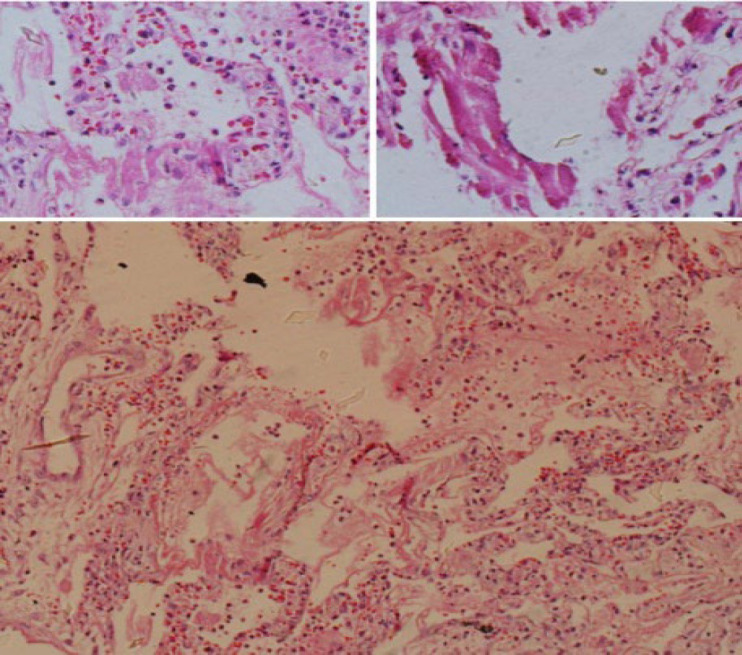
The lung biopsy specimen showing hyalin membrane

An 80-year-old man with a history of cerebrovascular accidents (C.V.A.) was admitted. The patient was then hospitalized and intubated in the ward for six days and then was transmitted to the Intensive care unit (I.C.U.) for another six days. The patient then died after 12 days of admission. Histopathological studies of the lung necropsies showed anthracosis, moderate interstitial lymphocytic pneumonitis, hyaline membrane, viral cytopathic effect, moderate interstitial fibrosis confirmed by trichrome stain, and no significant finding using iron-stained tissues (Figure 1).

**Case 2**

A 72-year-old woman was hospitalized for five days (3 days in I.C.U.) and died due to COVID-19 after five days of admission. The patient had no prior history of comorbidity and was not intubated during her hospitalization. Her necropsy findings suggested anthracosis, moderate interstitial lymphocytic pneumonitis, mild interstitial fibrosis, and negative iron stain findings.

**Case 3**

An 85-year-old woman with chronic obstructive pulmonary disease (COPD) was admitted to the infectious ward. The patient was admitted to the I.C.U. after one day of hospitalization, during which she was intubated and died after seven days. Histopathological studies showed marked interstitial lymphocytic pneumonitis, focal hyaline membrane, mild interstitial fibrosis confirmed by trichrome stain, and negative iron stain finding. 

**Case 4**

A 52-year-old woman with a history of hypertension was hospitalized for roughly a month. After 24 days of admission, she died of COVID-19. She was not intubated during her hospitalization. Her necropsy studies demonstrated mild interstitial lymphocytic pneumonitis, normal trichrome, and iron stain findings.

**Case 5**

An 80-year-old man with COVID-19 was admitted and hospitalized. He received no intubation and was not hospitalized in the intensive care unit. After his 8-day hospitalization, he died of COVID-19. According to the histopathologic findings, he presented with mild interstitial lymphocytic pneumonitis, anthracosis, focal neutrophilia, acute pneumonitis, and negative trichrome and iron stain results.

**Case 6**

An 85-year-old man with a history of cirrhosis was admitted. He was hospitalized for two days (with intubation) and died after. His necropsy studies suggested interstitial lymphocytic pneumonitis, anthracosis, negative trichrome, and iron stains findings.

**Case 7**

**Fig. 2:**
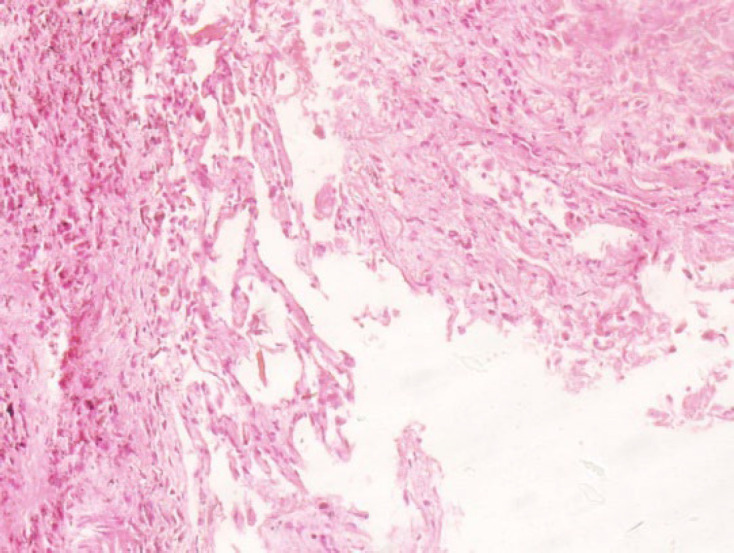
The lung biopsy specimen showing interstitial fibrosis and chronic inflammation

A 67-year-old man with prior C.V.A. history was hospitalized. He was intubated during his hospitalization and died 11 days after his admission. His histopathologic studies showed severe interstitial lymphocytic pneumonitis, intra-alveolar exudate with organization, bronchiolitis (BOOP pattern), intra-alveolar hemorrhage, focal iron stain finding, and mild interstitial fibrosis using trichrome stain (Figure 2).

**Case 8**

An 86-year-old woman was admitted and hospitalized, during which she was intubated. She had a history of hypertension and chronic kidney disease (CKD). Then, she died of COVID-19 after four days of non-ICU hospitalization. Her pathologic findings included interstitial lymphocytic pneumonitis, interstitial fibrosis, interstitial fibrosis (using trichrome stain), anthracosis, and negative iron stain findings.

**Case 9**

**Fig. 3:**
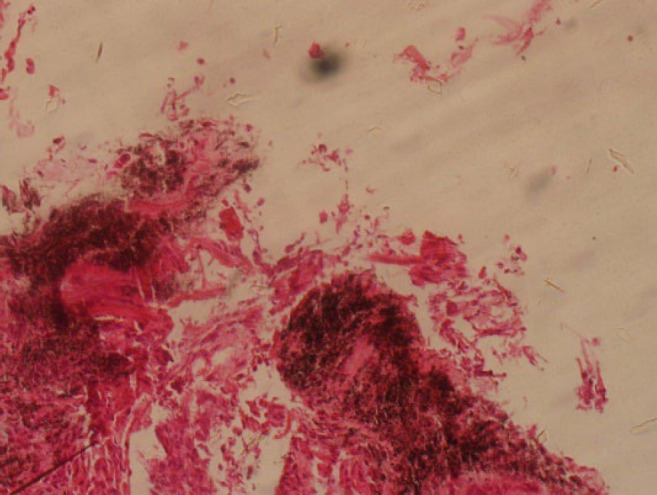
The lung biopsy specimen showing anthracosis and interstitial fibrosis

A 70-year-old man was hospitalized (35 days in I.C.U.) and died due to COVID-19 after 54 days of admission. The patient had a prior history of hypertension and was intubated during her hospitalization. His necropsy findings suggested anthracosis, marked interstitial pneumonitis, mild interstitial fibrosis confirmed by trichrome, and negative iron stains results. (Figure 3).

**Case 10**

A 71-year-old woman was hospitalized. After 28 days of admission and hospitalization in the I.C.U., she died of COVID-19. She was intubated during her hospitalization. She presented wih anthracosis, fibrosis, and alveolar hemorrhage in her necropsy studies.

**Case 11**

A 64-year-old man with a prior history of rheumatoid arthritis, COPD, and hypertension was admitted to I.C.U. During hospitalization, he was not intubated. After one day of hospitalization, he died due to COVID-19 confirmed by the tests. The histopathologic studies showed adipose connective tissue in pleura, mild pleuritis, and viral lymphocytes.

## Discussion

Atypical COVID-19 manifestations, including classic acute respiratory distress syndrome, can lead to the design and conducting of such histopathologic studies, which pave the way for management of the patients with more severe pulmonary involvement. Results of the necropsy studies from the patients showed interstitial lymphocytic pneumonitis in most of the cases, with severities ranging from mild to moderate and severe in some cases, respectively. 

A pleural necropsy taken from one of the cases reported mild pleuritis with lymphocytic infiltration. Consistent with this study, in a case report in 2020, Oleynick discussed a 48-year-old male presented with symptoms of pleurisy as the initial symptoms of COVID-19, which eventually, after ruling out for other hypoxemia and chest pain-related paraclinical assessments was diagnosed with viral pleuritis. As COVID-19 can present with various clinical pictures, non-classic presentations seem probable and should also be considered (6).

In 7 cases, Anthracosis and, in one case, anthracosis with fibrosis was evident. Since these are chronic histological reactions, they are not associated with COVID-19 and are probably related to the previous underlying conditions. However, in a similar previous case study by Kooranifar *et al.,* a high percentage of anthracosis was reported in the necropsy studies of patients, and it was proposed as a potential risk factor for COVID-19 mortality (7).

Hyaline membrane, a presentation of an early-phase acute respiratory distress syndrome, was reported in two patients. This finding has been also reported by Kooranifar *et al.* and Wang *et al.* (7, 8).

In case number 7, a 67-year-old man, severe interstitial lymphocytic pneumonia with intra-alveolar exudate with organization, lithiasis, bronchiolitis pattern (BOOP) along intra-alveolar hemorrhage, and mild fibrosis were described. Based on the clinical, paraclinical, and autopsy studies in the literature, organizing pneumonia (O.P.) is a common event in early COVID-19 respiratory complications (9). In a similar report by Copin M-C *et al.*, lung necropsies of six COVID-19 patients who died three weeks after the onset of symptoms also showed fibrinous organizing pneumonia (10).

Diffuse alveolar damage is the dominant pattern in COVID-19 pathology. Complement-mediated endothelial damage leads to immune-mediated vascular damage, vascular thrombosis, and pulmonary embolism in complicated patients. The vascular and thrombotic profile of COVID-19 pneumonia, which involves small and medium-sized vessels, has been well supported throughout the literature (11-14). In a study done by Yasmine Abourida *et al.*, in which lung mini-thoracotomy of three patients with post-COVID ARDS was histopathologically studied, diffuse alveolar damage with hyaline membrane along with plurifocal fibrin microthrombus and vascular congestion was reported. COVID-19-associated hypoxia and ventilation/perfusion mismatch were suggested as the two main factors behind the ARDS incidence. However, increased alveolar thickness and hyaline membrane can induce hypoxia, and other factors can also be involved. Direct pulmonary parenchymal invasion, cytopathic changes, and alveolar collapse lead to a reduction in the ventilation-perfusion ratio (15). Consistent with this study, as previously mentioned, a hyaline membrane was also evident in two patients. However, no clear signs of microthrombi or vascular pathologies were observed, and evidence of alveolar hemorrhage was seen in one case. Also, in a study performed by Bruce-Brand et al., histopathologic findings were described which were heterogeneous features including diffuse alveolar damage, diffuse fibrin thrombosis in pulmonary arteries, pulmonary infarctus, bronchopneumonia, and organizing pneumonia similar to our study. Other findings included type 2 pneumocytic hyperplasia, intra-alveolar macrophage, and squamous metaplasia (16).

Comorbidities, especially in older adults, is an important component leading to poor outcome in COVID-19 patients. In this study, hypertension, COPD, and liver and kidney diseases were among the most prevalent comorbidities in these patients. Similarly, in the Borczuk *et al.* study, comorbid conditions such as diabetes, hypertension, COPD, and ischemic heart diseases were reported in almost all of the patients (17). In addition, in a study performed by Menter *et al.*, who investigated autopsies of 21 COVID-19 patients, d capillary congestion, microthrombus, superimposed pneumonia, pulmonary emboli, and vasculitis were observed. They concluded less tolerance of COVID-19 by older men with pre-existing obesity, hypertension, diabetes, and vascular diseases (18).

## Conclusion

Atypical COVID-19 manifestations, including classic acute respiratory distress syndrome, may result in designing and conducting such histopathologic studies, paving the way for management of the patients with more severe pulmonary involvement. The results of the necropsy studies from the patients showed interstitial lymphocytic pneumonitis varied from mild to moderate in most of the cases, and severe in some cases. It also showed several other histopathologic findings. Necessary precautions should be taken in the management of the patients.

## Ethics Committee Approval Code

 The Ethics Committee of Kurdistan University of Medical Sciences (IR.MUK. REC.1399.024) reviewed and approved this study (https://ethics.research.ac.ir/EthicsProposalView.php?id=132918). For all patients, written informed consent was collected from their family members. All the relevant guidelines and regulations were met during the research.

## Conflict of Interest

The authors declare that they have no competing interests.

## Authors' Contributions

- B.M.G and B.N. conceived and designed the study. – R.R.D., M.H., and M.R. analyzed and interpreted the data and drafted the manuscript. – B.N. K.M. and B.M.G were involved in the study tool's composition, collecting data, supervising the research process, and critically revising and reviewing the manuscript. All the authors read and approved the final manuscript**. **
